# A Study of Logistic Regression for Fatigue Classification Based on Data of Tongue and Pulse

**DOI:** 10.1155/2022/2454678

**Published:** 2022-03-05

**Authors:** Yu Lin Shi, Tao Jiang, Xiao Juan Hu, Ji Cui, Long Tao Cui, Li Ping Tu, Xing Hua Yao, Jing Bin Huang, Jia Tuo Xu

**Affiliations:** ^1^Department of Basic Medical College, Shanghai University of Traditional Chinese Medicine, 1200 Cailun Road, Pudong, Shanghai, China; ^2^Shanghai Collaborative Innovation Center of Health Service in Traditional Chinese Medicine, Shanghai University of Traditional Chinese Medicine, 1200 Cailun Road, Pudong, Shanghai, China

## Abstract

**Methods:**

The Tongue and Face Diagnosis Analysis-1 instrument and Pulse Diagnosis Analysis-1 instrument were used to collect the tongue image and sphygmogram of the subhealth fatigue population (*n* = 252) and disease fatigue population (*n* = 1160), and we mainly analyzed the tongue and pulse characteristics and constructed the classification model by using the logistic regression method.

**Results:**

The results showed that subhealth fatigue people and disease fatigue people had different characteristics of tongue and pulse, and the logistic regression model based on tongue and pulse data had a good classification effect. The accuracies of models of healthy controls and subhealth fatigue, subhealth fatigue and disease fatigue, and healthy controls and disease fatigue were 68.29%, 81.18%, and 84.73%, and the AUC was 0.698, 0.882, and 0.924, respectively.

**Conclusion:**

This study provided a new noninvasive method for the fatigue diagnosis from the perspective of objective tongue and pulse data, and the modern tongue diagnosis and pulse diagnosis have good application prospects.

## 1. Introduction

Fatigue refers to physical tiredness with lack of energy or mental exhaustion with lack of concentration. It can be divided into physical fatigue and mental fatigue [1]. Fatigue is the first cause of subhealth and is one of the most common symptoms in primary care, and it is experienced by many patients with chronic hepatitis [2, 3], depression [4], and various types of cancers [5]. Subhealth and a wide variety of diseases are associated with different degrees of fatigue with a negative effect on people's life. With the improvement of general medical care and living standard, fatigue is more and more known by people; however, due to the lack of objective diagnostic evidence, there is still no reliable and stable evaluation method to distinguish disease fatigue and subhealth fatigue.

A large number of clinical practices and studies have shown that tongue and pulse can reflect the overall state of body [6]. Tongue and pulse of fatigue people have their own characteristics. Studies have shown that the tongue of patients with brain fatigue is usually dull, slow in movements, weak, or difficult to stretch [7]. In the physical examination population, the tongue of fatigue population has certain specificity in tongue body color, tongue coating color, and tongue shape. Fatigue is closely related to the tooth mark tongue, and the degree of fatigue is positively related to the tooth mark area [8]. The tongue of patients with fatigue syndrome has obvious characteristics of stasis, mainly purple tongue, petechiae or ecchymosis tongue, sublingual varices, and white thick tongue coating [9]. In patients with chronic fatigue syndrome in Hong Kong, the tongue body is usually light fat and dull, tongue coating is thin white, or thin greasy, and the pulse is usually deep and thin [10].

Intelligent diagnosis of TCM is a new research field in recent years, and it meets the trend that TCM diagnosis methods are developing gradually towards intelligence and potential application in clinical practice [11, 12]. In recent research of tongue diagnosis and pulse diagnosis, new diagnosis systems are adopted to collect and analyze clinical data related to disease, and machine learning methods such as Artificial Neural Network [13, 14], Support Vector Machine(SVM) [15, 16], and KNN [17] are used to establish the corresponding diagnosis model, which can effectively assist doctor on the diagnosis of disease. In recent years, there have been more and more objectified and standardized studies on fatigue based on tongue diagnosis and pulse diagnosis [18–20].

Based on the modern research of tongue diagnosis and pulse diagnosis, this study aims to explore the distribution rules of tongue and pulse data in disease fatigue and subhealth fatigue and evaluate the contribution rate to fatigue diagnosis through modeling, so as to provide a new reference for convenient and noninvasive methods of fatigue diagnosis, and if an objective evaluation method based on tongue and pulse data can be established, it will play an important role in the clinical diagnosis of fatigue.

## 2. Methods

### 2.1. Study Design

**Figure 1:**
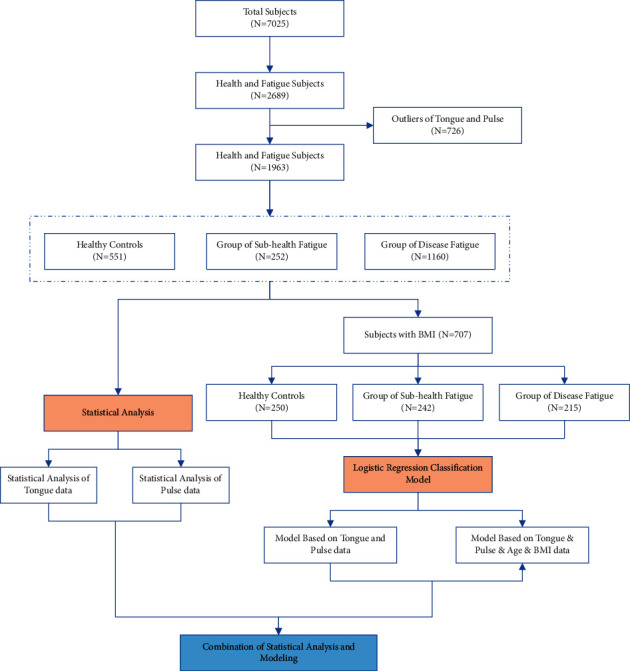
Overall flow diagram.

A total of 7,025 subjects were collected from January 2015 to December 2018 in the medical examination center of Shuguang Hospital Affiliated to Shanghai University of Traditional Chinese Medicine, collecting their Western medicine physical examination index and tongue and pulse data of TCM. The 7,025 subjects were divided into healthy controls (*n* = 799), a subhealth fatigue group (*n* = 361), and a disease fatigue group (*n* = 1529). After excluding the outliers with extreme values in tongue or pulse data, there were 551, 252, and 1,160 subjects in healthy controls, the subhealth fatigue group, and the disease fatigue group, respectively. The overall flow diagram of the study is shown as Figure 1.

### 2.2. Diagnostic Criteria

Health and subhealth of each individual were determined using the Health Status Assessment Questionnaire H20 Scale [21] and the Information Record Form of Four Diagnosis of TCM [22] (Copyright No. 2016Z11L025702) which were designed by the Sub-Health Research Group. Excluding the disease population, the population with a score between 60 and 79 on the H20 scale was the subhealth population, and the population with a score between 80 and 100 on the H20 scale was healthy controls. The diagnostic criteria of disease are shown in Table 1.

Disease was diagnosed by four well-trained clinicians according to the abovementioned diagnostic criteria of Western medicine. The Information Record Scale of Four Diagnosis of TCM and H20 scale were used to select fatigue population, and people with “fatigue” symptom in the two scales were judged as the fatigue population.

### 2.3. Tongue Diagnosis and Pulse Diagnosis Instruments

**Figure 2:**
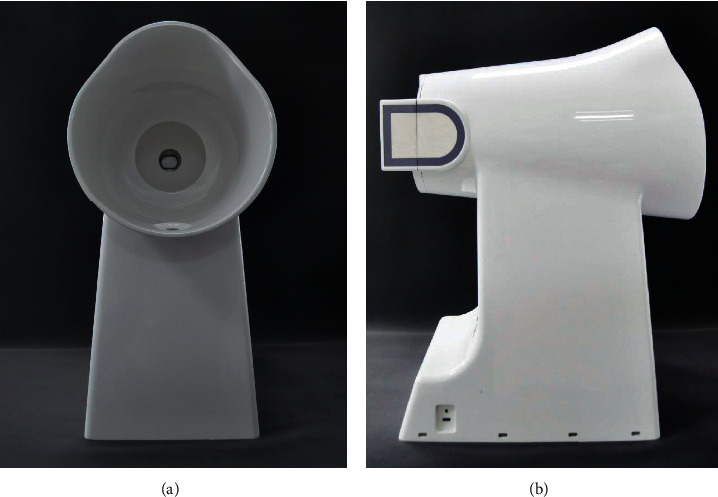
TFDA-1 tongue and face diagnosis instrument. (a) Front view. (b) Profile view.

**Figure 3:**
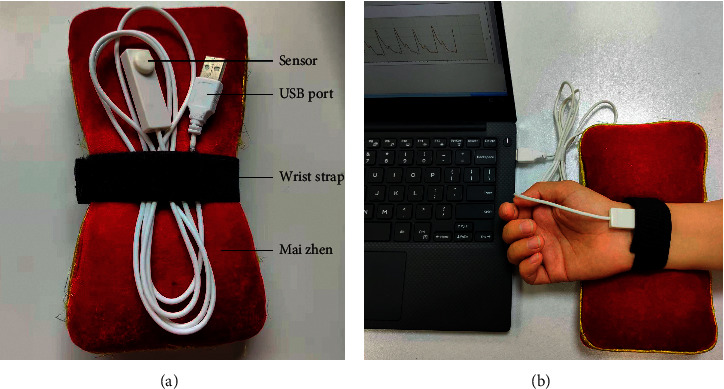
PDA-1 pulse diagnosis instrument and the corresponding collection picture. (a) PDA-1 pulse diagnosis instrument. (b) Real picture of pulse acquisition.

The TFDA-1 tongue and face diagnosis instrument (Patent no. 2018SR033451) [27] and PDA-1 pulse diagnosis instrument (Patent no. ZL201620157027.6) [28] are shown in Figures 2 and 3; they were used for data collection. The tongue was imaged by using a video camera (Nikon 1 J5) with a fixed-focal lens which has 12 megapixels, and the picture resolution is 5568*∗*3712. The color rendering index of light source was 96, and color temperature was around 5,000–6,500 K. The indices of the tongue image were from color spaces of RGB, HSI, Lab, and YCrCb. The prefix TB represented the tongue body index, and TC represented the tongue coating index. The PDA-1 pulse diagnosis instrument uses a pressure sensor (model: HK-2000H). Each of the indices of the tongue and pulse has its meaning [11, 13]. In our research, the normal range of L value was 0–255, and in order to better observe the continuity of the trend of data changes and find the data rules and real differences, we rotated the axis of H value by 180 degrees according to the law.

### 2.4. Data Analysis

SPSS 25.0 was used for statistical analysis. The normal distribution measurement data were expressed as “ Mean ± SD”. Nonnormal distribution data were expressed as quartiles expressed as “ Median (P25, P75).” Analysis of Variance (ANOVA) was performed for normality and homogeneity data among groups, the Kruskal–Wallis H test was performed for nonnormal distribution data, and GraphPad Prism Version 8.0 was used for the violin plot. Test level was *α* = 0.05, and a *P* value <0.05 (2 tailed) was considered statistically significant.

### 2.5. Modeling

Logistic regression analysis was performed for factors with statistical significance by ANOVA or the Rank Sum Test. Logistic regression is often used in data mining, automatic disease diagnosis, economic prediction, and others, and the accuracy of decision can be improved by adjusting the parameters of the regression model [29, 30]. The evaluation indices of the model were accuracy, sensitivity, and specificity, as well as ROC curves. They were defined as follows:

(1) $$\begin{array}{c} \text{Accuracy}=\frac{\text{TP}+\text{TN}}{\text{TP}+\text{TN}+\text{FP}+\text{FN}}\times 100\%,\\ \text{Sensitivity}=\frac{\text{TP}}{\text{TP}+\text{FN}}\times 100\%,\\ \text{Specificity}=\frac{\text{TN}}{\text{TN}+\text{FP}}\times 100\%. \end{array}$$

In the abovementioned formulas, TP represents the true positive rate, TN represents the true negative rate, FP represents the false positive rate, and FN represents the false negative rate.

## 3. Results

### 3.1. The Baseline Characteristics of Studied Participants

**Figure 4:**
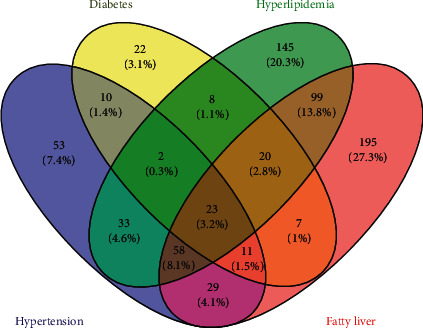
Distribution of main diseases in the group of disease fatigue.

The main diseases in the disease fatigue group were hypertension, diabetes, hyperlipidemia, and fatty liver, and their distribution is shown in Figure 4. The numbers and percentage in Figure 4 represent the number of patients and the ratio of the number of patients with the disease to the total number of patients, and the overlapping part represents the number and percentage of patients suffering from multiple diseases at the same time.  Table 2 shows the general result of the healthy controls, the group of subhealth fatigue, and the group of disease fatigue.

The statistical result showed that compared with the healthy controls, there were significant differences in age and BMI between the group of disease fatigue and the subhealth fatigue (*P* < 0.01).

### 3.2. Statistical Analysis of Tongue Indices

Table 3 shows the statistical analysis result of distribution of the characteristic parameters of the tongue body and tongue coating among the healthy controls, the group of subhealth fatigue, and the group of disease fatigue.

**Figure 5:**
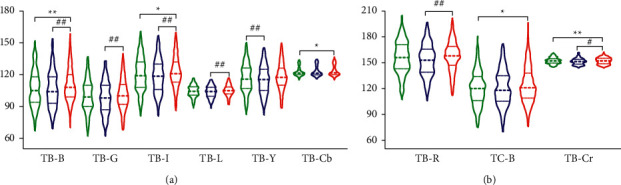
Violin plots of the tongue characteristic parameters of the three groups.

In order to observe the distribution trend of data more clearly, the violin plots of selected parameters of tongue body and tongue coating with statistical significance were drawn as shown in Figure 5.

The main results of tongue indices were as follows: (1) Comparing among the three groups, the changes of TB indices in the group of subhealth fatigue and disease fatigue were more significant than those in the TC indices. (2) Index difference was more significant between the group of disease fatigue and subhealth fatigue. (3) Several indices (TB-B, TB-R, TB-G, TC-B, TB-I, TB-Y, TB-L, TB-Cb, and TB-Cr) in the healthy controls were between the two fatigue groups. It reflected that the two groups of fatigue people had different tendencies in the changing nature of the tongue.

### 3.3. Statistical Analysis of Pulse Indices

Table 4 shows the statistical analysis result of the distribution of pulse characteristic parameters in healthy controls, the group of subhealth fatigue, and the group of disease fatigue.

**Figure 6:**
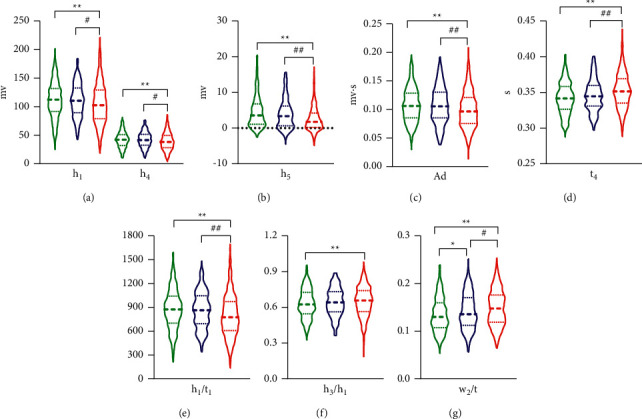
Violin plots of the pulse characteristic parameters of the three groups.

Figure 6 shows the violin plots of selected parameters of pulse characteristic with statistical significance.

The main result of pulse feature parameters showed that *t*_1_, *t*_2_, *t*_3_, *t*_4_, *h*_1_, *h*_4_, *h*_5_, *w*_1_, *w*_2_, *w*_1_/*t*, *w*_2_/*t*, *h*_1_/*t*_1,_*h*_3_/*h*_1_, As, and Ad had significant statistical differences between the group of disease fatigue and healthy controls (*P* < 0.05, *P* < 0.01), *t*_4_ had significantly statistical differences between the group of subhealth fatigue and the healthy controls (*P* < 0.05), and *t*_1_, *h*_1_, *h*_4_, *h*_5_, *h*_1_/*t*_1_, Ad, *w*_1_, *w*_2_, *w*_1_/*t*, and *w*_2_/*t* had significantly statistical differences between the group of subhealth fatigue and the disease fatigue (*P* < 0.05, *P* < 0.01). The main characteristic of result was that the group of subhealth fatigue and disease fatigue showed a gradual increasing tendency in each parameter compared with the healthy controls, and it reflected that the two groups of fatigue people had a consistent tendency in the changing nature of pulse. In addition, the changes of pulse feature in the group of disease fatigue were more significant than those in the group of subhealth fatigue.

### 3.4. Modeling Results

Logistic regression was used to establish a classification model based on tongue and pulse data of the three groups. The classification result is shown in Table 5.

**Figure 7:**
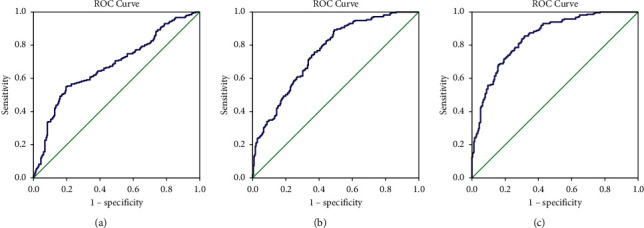
ROC curves of the classification model based on tongue and pulse data. (a) Healthy and subhealth fatigue. (b) Subhealth and disease fatigue. (c) Healthy and disease fatigue.

The ROC curves are shown in Figure 7.

In addition, the classification model was reconstructed after adding BMI and age into tongue and pulse data.  Table 6 shows the classification result of the reconstructed model.

**Figure 8:**
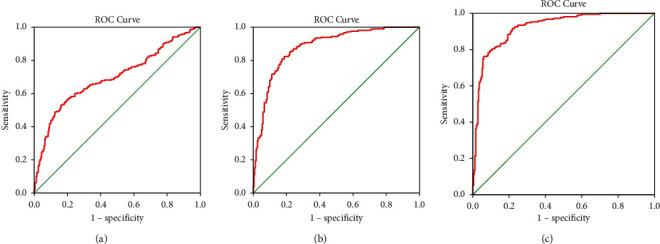
ROC curves of the classification model based on tongue and pulse data and BMI and age. (a) Healthy and subhealth fatigue. (b) Subhealth and disease fatigue. (c) Healthy and disease fatigue.

The ROC curves are shown in Figure 8.

The research result showed that tongue and pulse data had a good classification effect on healthy controls and disease fatigue, followed by healthy controls and subhealth fatigue. After adding BMI and age, both of the model accuracy and ROC curves were improved. BMI and age are convenient and noninvasive data, which suggested that we could combine BMI and age with tongue and pulse data to improve the diagnostic accuracy of fatigue.

## 4. Discussion

In this study, the distribution trends of the objective tongue data were different between the subhealth fatigue population and the disease fatigue population. The study showed that TB-B, TB-R, TB-G, TC-B, TB-I, TB-Y, TB-L, TB-Cb, TB-Cr, TB-a, and TC-a were in an ascending order in the group of subhealth fatigue, healthy controls, and the group of disease fatigue, which indicated that disease fatigue people in general had more purple or red-purple tongue body and more white-greasy tongue coating. The tongue parameters of the subhealth fatigue population were lower than those of the healthy controls, while those of disease fatigue were higher than those of the healthy controls. Certain differences were found in tongue parameters of fatigue groups compared with the healthy controls; that is, subjects in the group of disease fatigue had darker tongue body and tongue coating and more yellow or brown tongue coating, which was more associated with excess syndrome, and the subjects in the group of subhealth fatigue had a pale red tongue body with white coating, which was more associated with the deficiency syndrome. The finding was consistent with the TCM theory that subhealth was manifested as decreased vitality, function, and adaptability, and disease was mostly due to the hyperactivity of evil spirits or dysfunction of the dysfunctional organs caused by phlegm [31], dampness [32], and blood stasis [33] and other pathological products. The result could help to distinguish subhealth fatigue and disease fatigue.

In our study, the pulse analysis result of the three groups showed that fatigue state can directly affect the changes of sphygmogram parameters, and the change had a consistent trend; so to say, the indices of disease fatigue were more abnormal and the differences were more significant compared to healthy controls, while between the group of subhealth fatigue and health controls, only *w*_2_/*t* had a statistical difference, and several indices had a significant difference between the group of subhealth fatigue and disease fatigue. As to the distribution trend of pulse indices, the group of subhealth fatigue was located between healthy controls and the group of disease fatigue. Studies have shown that pulse can directly reflect various cardiovascular functional states [34, 35], and the results of this study, to a certain extent, indicated that patients with disease fatigue had more severe functional decline and other abnormal changes in cardiovascular functions, such as left ventricular function, peripheral resistance, great artery compliance, wall elasticity, and blood viscosity. Since fatigue in the most serious case can cause sudden cardiac death, it was of great practical value to use a sphygmograph to detect fatigue in order to diagnose cardiovascular disease and help to guide the early intervention.

In this study, our focus was on whether tongue and pulse data or tongue and pulse combined with age and BMI could distinguish different fatigue states well and whether age and BMI affected tongue and pulse, but to what extent was not the focus of our study. Age and BMI are the basic information of human health and are closely related to disease. Studies have shown that there was a correlation between age and disease [36]; with the increase in age, the risk of disease gradually increased. BMI is an index of obesity which is closely related to health state, and studies have shown that the BMI combined circumference level can be used to assess the risk of coronary heart disease in diabetic patients [37]. Our actual research results also conform to this law, age and BMI combined with tongue and pulse data had a better effect on the classification of fatigue.

This study provided a noninvasive differential diagnosis method for the data-driven evaluation of different fatigue states based on the data of tongue and pulse, and modern tongue diagnosis and pulse diagnosis have good application prospects. The modern technique of tongue diagnosis and pulse diagnosis is simple and feasible. With the development of the information technology of tongue diagnosis and pulse diagnosis, a small, convenient, and movable tongue diagnosis and pulse diagnosis instrument has provided the possibility of family health monitoring. With the development of more wearable health products and more personal health data collection and use, the integration of tongue diagnosis and pulse diagnosis information with other health information can effectively judge fatigue and other health conditions and make early warning of diseases, and it also can effectively promote the development of Internet smart medical treatment and remote diagnosis and treatment and innovate and develop the intelligent diagnosis and treatment model of TCM. In the future, on the basis of multidisciplinary interaction, natural language interaction or graphical interface, multichannel intelligent human-computer interaction, data mining, and machine learning based on big data to achieve automated analysis, we can effectively improve the accuracy of diagnosis and treatment.

## 5. Conclusions

In this study, we successfully analyzed the tongue and pulse data characteristics and distribution trend of the fatigue and healthy population; at the same time, logistic regression modeling can realize the diagnosis of disease fatigue and subhealth fatigue to a certain extent. It provided a noninvasive differential diagnosis method for the data-driven evaluation of different fatigue states based on the data of the tongue and pulse.

## Figures and Tables

**Table 1:** Diagnostic criteria of disease.

Disease	Diagnostic criteria
Diabetes [23]	Fasting blood glucose ≥7.0 mmol/L and/or blood glucose at any point ≥7.8 mmol/L and/or blood glucose at two hours after meal ≥11.1 mmol/L
Hypertension [24]	Systolic blood pressure≥140 mmHg and/or diastolic blood pressure ≥90 mmHg
Hyperlipidemia [25]	TC ≥ 6.2 mmol/L and/or LDL-C ≥ 4.1 mmol/L and/or HDL-C ≥ 4.9 mmol/L and/or TG ≥ 2.3 mmol/L and/or non-HDL-C ≥ 1.55 mmol/L
Fatty liver disease [26]	Ultrasound examination

**Table 2:** General result (mean (SD) and median (P25, P75)).

Group		*N*	Male	Female	Age	BMI
			N (%)	N (%)	(Mean ± SD, year)	(Kg/m^2^)
Healthy controls		551	394 (71.5)	157 (28.5)	29.00 (25.00,35.00)	22.31 (20.55,24.51)
Subhealth fatigue		252	149 (59.1)	103 (40.9)	32.00 (28.00,37.00) ^*∗*^	22.39 (20.28,24.68)
Disease fatigue	Hypertension	219	160 (73.1)	59 (26.9)	46.00 (36.00,57.00) ^*∗∗*^^##^	25.30 (23.40,27.40) ^*∗∗*^^##^
	Diabetes	103	84 (81.6)	19 (18.4)	56.00 (45.00,63.00) ^*∗∗*^^##^	25.20 (23.50,27.70) ^*∗∗*^^##^
	Hyperlipidemia	388	272 (70.1)	116 (29.9)	43.50 (34.00,53.00) ^*∗∗*^^##^	24.90 (22.90,26.90) ^*∗∗*^^##^
	Fatty liver	442	334 (75.6)	108 (24.4)	45.00 (34.00,55.00) ^*∗∗*^^##^	26.20 (24.60,28.10) ^*∗∗*^^##^

Vs. healthy controls,  ^*∗*^*P* < 0.05, vs. healthy controls, *∗∗P* < 0.01, vs. subhealth fatigue, ^#^*P* < 0.05, vs. subhealth fatigue, ^##^*P* < 0.01.

**Table 3:** Statistical result of the tongue body and tongue coating (mean (SD) and median (P_25_, P_75_)).

Domain	Color space	Index	Healthy controls	Subhealth fatigue	Disease fatigue
			(*N* = 551)	(*N* = 252)	(*N* = 1160)
TB	RGB	TB-R	156.00(143.00,171.00)	153.00(139.00,165.75)	158.00(147.00,169.00)^##^
		TB-G	99.00(90.00,110.00)	98.00(87.00,110.00)	100.00(92.00,110.75)^#^
		TB-B	105.00(94.00,118.00)	104.00(93.00,118.00)	108.00(99.00,120.00) ^*∗∗*^^##^
	YCrCb	TB-Y	115.77(107.05,126.27)	115.40(105.14,125.09)	117.46(110.08,126.16)^##^
		TB-Cr	152.17(149.64,154.65)	151.31(148.37,153.84) ^*∗∗*^	152.21(148.82,155.17)^#^
		TB-Cb	121.46(119.99,124.21)	121.32(119.84,126.08)	121.74(120.00,127.46) ^*∗*^
	HSI	TB-H	176.27(169.98,179.15)	176.52(166.25,180.00)	175.16(162.12,178.32) ^*∗∗*^^##^
		TB-S	0.18(0.16,0.20)	0.18(0.15,0.20)	0.18(0.16,0.20)
		TB-I	119.00(108.00,132.00)	118.50(106.00,130.00)	121.00(113.00,132.00) ^*∗*^^##^
	Lab	TB-L	104.33(100.72,108.58)	104.08(99.70,108.05)	104.94(101.84,108.42)^##^
		TB-a	20.62(18.42,22.79)	20.49(18.11,22.68)	20.98(18.69,23.13)
		TB-b	4.91(1.64,6.59)	5.01(−0.17,6.87)	4.35(−1.82,6.23) ^*∗∗*^^#^
	Texture index	TB-CON	65.33(42.36,92.43)	66.07(40.86,97.57)	60.92(41.26,85.49)
		TB-ENT	1.19(1.09,1.27)	1.19(1.08,1.28)	1.17(1.08,1.26)
		TB-ASM	0.08(0.07,0.10)	0.08(0.07,0.10)	0.08(0.07,0.10) ^*∗*^
		TB-MEAN	0.02(0.02,0.03)	0.02(0.02,0.03)	0.02(0.02,0.03) ^*∗*^

TC	RGB	TC-R	152.00(17.65)	149.56(17.84)	152.44(16.62)
		TC-G	115.00(103.00,126.00)	114.50(103.00,127.00)	114.00(104.00,126.00)
		TC-B	120.00(106.00,134.00)	118.00(105.25,135.00)	121.00(109.00,138.00)*∗*
	YCrCb	TC-Y	124.63(115.36,134.38)	123.65(113.84,134.00)	124.44(116.39,134.07)
		TC-Cr	143.88(141.46,146.59)	143.15(140.51,145.71)*∗*	143.95(140.29,147.04)
		TC-Cb	123.25(121.65,126.17)	123.25(121.64,128.76)	123.76(121.83,129.82)*∗∗*
	HSI	TC-H	177.27(167.16,181.53)	177.39(156.48,182.40)	175.23(151.57,180.00)*∗∗*^##^
		TC-S	0.12(0.10,0.14)	0.12(0.09,0.14)	0.12(0.10,0.14)
		TC-I	129.00(117.00,140.00)	128.00(115.00,140.00)	129.00(119.00,140.00)
	Lab	TC-L	108.41(104.62,112.06)	108.11(104.12,112.09)	108.28(105.06,112.02)
		TC-a	13.10(2.78)	12.71(2.78)	13.27(2.75)
		TC-b	3.17(0.03,4.86)	3.22(-1.97,4.95)	2.63(−3.34,4.68) ^*∗*^
	Area index	perAll	0.50(0.41,0.67)	0.53(0.41,0.76)	0.49(0.38,0.79)
		perPart	1.11(1.04,1.25)	1.08(1.03,1.22)	1.10(1.02,1.21)
	Texture index	TC-CON	82.23(57.65,115.59)	88.97(58.76,125.73)	82.58(56.88,114.81)
		TC-ENT	1.25(1.16,1.32)	1.27(1.17,1.34)	1.25(1.16,1.33)
		TC-ASM	0.07(0.06,0.08)	0.07(0.06,0.08)	0.07(0.06,0.09)
		TC-MEAN	0.03(0.02,0.03)	0.03(0.02,0.03)	0.03(0.02,0.03)

Vs. healthy controls,  ^*∗*^*P* < 0.05, vs. healthy controls,  ^*∗∗*^*P* < 0.01, vs. subhealth fatigue, ^#^*P* < 0.05, vs. subhealth fatigue group, ^##^*P* < 0.01.

**Table 4:** Statistical result of pulse characteristic parameters (mean (SD) and median (P_25_, P_75_)).

Index	Healthy controls	Subhealth fatigue	Disease fatigue
	(*N* = 551)	(*N* = 252)	(*N* = 1160)
*t*_1_(s)	0.13(0.12,0.14)	0.13(0.12,0.14)	0.13(0.12,0.14)*∗∗*^##^
*t*_2_(s)	0.22(0.21,0.24)	0.22(0.21,0.24)	0.23(0.22,0.24)*∗∗*^##^
*t*_3_(s)	0.26(0.25,0.27)	0.26(0.25,0.27)	0.26(0.25,0.28)*∗∗*^#^
*t*_4_(s)	0.34(0.33,0.36)	0.34(0.33,0.36)	0.35(0.34,0.37)*∗∗*^##^
*t*_5_(s)	0.40(0.39,0.42)	0.41(0.39,0.42)	0.41(0.39,0.42)
*h*_1_(mv)	112.37(91.47,131.98)	110.54(89.53,132.73)	102.70(79.10,129.20)*∗∗*^#^
*h*_2_(mv)	76.26(57.64,96.64)	76.52(60.46,95.92)	72.92(53.31,96.65)
*h*_3_(mv)	68.33(53.88,88.51)	69.70(55.68,87.59)	65.87(48.83,86.66)
*h*_4_(mv)	42.20(32.03,51.17)	41.58(32.52,51.59)	38.25(28.18,49.68)*∗∗*^#^
*h*_5_(mv)	3.54(1.08,6.80)	3.35(0.66,6.18)	1.76(0.18,4.22)*∗∗*^#^
*w*_1_(*s*)	0.16(0.13,0.19)	0.17(0.14,0.19)	0.17(0.14,0.20)*∗∗*
*w*_2_(*s*)	0.10(0.09,0.13)	0.11(0.09,0.14)	0.12(0.09,0.15)*∗∗*^#^
*w*_1_/*t*	0.20(0.16,0.22)	0.20(0.18,0.23)	0.21(0.18,0.24)*∗∗*^#^
*w*_2_/*t*	0.13(0.11,0.16)	0.14(0.11,0.17)*∗*	0.15(0.12,0.18)*∗∗*^#^
*h*_1_/*t*_1_	874.36(701.84,1041.02)	862.66(693.70,1046.20)	775.13(607.61,972.90)*∗∗*^##^
*h*_3_/*h*_1_	0.62(0.55,0.73)	0.64(0.56,0.73)	0.66(0.56,0.74)*∗∗*
*h*_4_/*h*_1_	0.38(0.08)	0.38(0.08)	0.37(0.08)
As(mv·s)	0.20(0.03)	0.21(0.03)	0.21(0.03)*∗∗*^#^
Ad(mv·s)	0.11(0.09,0.13)	0.11(0.09,0.13)	0.10(0.08,0.12)*∗∗*^##^
*t*(*s*)	0.82(0.75,0.90)	0.82(0.77,0.90)	0.82(0.75,0.90)

Vs. healthy controls, *∗P* < 0.05, vs. healthy controls, *∗∗P* < 0.01, vs. subhealth fatigue, ^#^*P* < 0.05, vs. subhealth fatigue, ^##^*P* < 0.01.

**Table 5:** Classification result of the model based on tongue and pulse data.

Model	AUC	Accuracy (%)	Sensitivity (%)	Specificity (%)
Healthy controls and subhealth fatigue	0.678	65.70	73.60	57.44
Subhealth and disease fatigue	0.759	67.40	73.55	60.47
Healthy controls and disease fatigue	0.847	76.30	80.40	71.63

**Table 6:** Classification result of the model based on tongue and pulse data and BMI and age.

Model	AUC	Accuracy (%)	Sensitivity (%)	Specificity (%)
Healthy controls and subhealth fatigue	0.698	68.29	80.80	55.37
Subhealth and disease fatigue	0.882	81.18	83.06	79.07
Healthy controls and disease fatigue	0.924	84.73	87.60	81.40

## Data Availability

The datasets generated and analyzed during the current study are not publicly available due to the confidentiality of the data, which is an important component of the National Key Technology R & D Program of the 13th Five-Year Plan (no. 2017YFC1703301) in China, but are available from the corresponding author on reasonable request.

## References

[1] Persson P. B., Bondke Persson A. (2016). Fatigue. *Acta Physiology (Oxford)*.

[2] Golabi P., Sayiner M., Bush H., Gerber L. H., Younossi Z. M. (2017). Patient-reported outcomes and fatigue in patients with chronic hepatitis C infection. *Clinics in Liver Disease*.

[3] Swain M. G., Jones D. E. J. (2019). Fatigue in chronic liver disease: new insights and therapeutic approaches. *Liver International*.

[4] Chung K. F., Yu Y. M., Yeung W. F. (2015). Correlates of residual fatigue in patients with major depressive disorder: the role of psychotropic medication. *Journal of Affective Disorders*.

[5] Ebede C. C., Jang Y., Escalante C. P. (2017). Cancer-related fatigue in cancer survivorship. *Medical Clinics of North America*.

[6] Thirunavukkarasu U., Umapathy S., Krishnan P. T., Janardanan K. (2020). Human Tongue thermography could be a prognostic tool for prescreening the type II diabetes mellitus. *Evidence Based Complementary Alternative Medicine*.

[7] Li N. M., Li S. W., Liu S., Wang C. Y., Cui Z. C. (2014). Clinical study of brain fatigue of tongue picture[J]. *Lishizhen Medicine and Materia Medica Research*.

[8] Ding T., Feng L., Rong L., Danxi L., Syria Y. Y., Ying T. Tongue inspection on fatigue.

[9] Chunyan W. Observation of tongue and pulse representation in 147 cases of fatigue syndrome.

[10] Yiu Y. M., Qiu M. Y. (2005). A preliminary epidemiological study and discussion on traditional Chinese medicine pathogenesis of chronic fatigue syndrome in Hong Kong. *Journal of Chinese Integrative Medicine*.

[11] Luo Z. Y., Cui J., Hu X. J., Tu L. P., Liu H. D., Jiao W. (2018). A study of machine-learning classifiers for hypertension based on radial pulse wave. *Biomed Research International*.

[12] Hu X. J., Zhang L., Xu J. T., Liu B. C., Wang J. Y., Hong Y. L. (2018). Pulse wave cycle features analysis of different blood pressure grades in the elderly. *Evidence Based Complement Alternative Medicine*.

[13] Li J., Yuan P., Hu X. (2021). A tongue features fusion approach to predicting prediabetes and diabetes with machine learning. *Journal of Biomedical Informatics*.

[14] Tang A. C. Y., Chung J. W. Y., Wong T. K. S. (2012). Digitalizing traditional Chinese medicine pulse diagnosis with artificial neural network. *Telemedicine and E-Health*.

[15] Zhang J., Xu J., Hu X., Chen Q., Tu L., Huang J. (2017). Diagnostic method of diabetes based on support vector machine and tongue images. *Biomed Research International*.

[16] Hu M. C., Lan K. C., Fang W. C. (2019). Automated tongue diagnosis on the smartphone and its applications. *Computer Methods and Programs in Biomedicine*.

[17] Zhang B., Wang X., You J., Zhang D. (2013). Tongue color analysis for medical application. *Evidence Based Complement Alternative Medicine*.

[18] Kung Y. Y., Kuo T. B. J., Lai C. T., Shen Y. C., Su Y. C., Yang C. C. H. (2021). Disclosure of suboptimal health status through traditional Chinese medicine-based body constitution and pulse patterns. *Complementary Therapies in Medicine*.

[19] Shi H. Z., Fan Q. C., Gao J. Y. (2017). Evaluation of the health status of six volunteers from the mars 500 project using pulse analysis. *Chinese Journal of Integrative Medicine*.

[20] Wang L. L., Zhang X. Y., Peng M. (2019). Objective analysis of complexion and tongue color in patients with chronic fatigue syndrome. *Shandong Medical Journal*.

[21] Zhu H. H., Zhang Z. F., Chen X., Fei Z. F., Xu J. T. Design and evaluation of the simple health assessment questionnaire H2O.

[22] Zhang J. F., Xu J. T., Tu L. P., Zhang Z. F., Hu X. J., Cui J. (2017). Study on the characteristics of sub-health symptoms and TCM syndrome patterns distribution in 1 754 non-disease population. *Chinese Journal of Integrative Medicine*.

[23] Chinese Diabetes Society (2018). Guidelines for the prevention and control of type 2 diabetes in China. *Chinese Journal of Practical Internal Medicine*.

[24] Liu J. (2020). Highlights of the 2018 Chinese hypertension guidelines. *Clinical Hypertension*.

[25] Chen Y., Chen Y. B., Tao R. T. (2017). Interpretation of “guideline for prevention and treatment of dyslipidemia in Chinese adults in 2016. *Chinese Journal of Practical Internal Medicine*.

[26] Fatty Liver and Alcoholic L:iver Disease Group, Hepatology Society, Chinese Medical Association (2001). Diagnostic criteria for nonalcoholic fatty liver disease (draft). *Chinese Journal of Hepatology*.

[27] Huang J. B., Xu J. T., Zhang Z. F., Tu L. P., Cui J., Hu X. J. (2018). Influence of “daily rhythm” factors on tongue image characteristics of healthy people. *China Journal of Traditional Chinese Medicine and Pharmacy*.

[28] Cui J., Tu L. P., Zhang J. F., Hu X. J., Huang J. B., Wang G. L. (2018). Research on pulse graph characteristics of 1 720 cases with different health status and age gradient. *Shanghai Journal of Traditional Chinese Medicine*.

[29] Bucur E., Danet A. F., Lehr C. B., Lehr E., Nita-Lazar M. (2017). Binary logistic regression-instrument for assessing museum indoor air impact on exhibits. *Journal of the Air & Waste Management Association*.

[30] Zhang K., Geng W., Zhang S. (2018). Network-based logistic regression integration method for biomarker identification. *BMC Systems Biology*.

[31] Sun D. Z., Xu L., Wei P. K., Liu L., He J. (2007). Syndrome differentiation in traditional Chinese medicine and E-cadherin/ICAM-1 gene protein expression in gastric carcinoma. *World Journal of Gastroenterology*.

[32] Zhang N., Li C., Guo Y., Wu H. C. (2020). Study on the intervention effect of qi gong wan prescription on patients with phlegm-dampness syndrome of polycystic ovary syndrome based on intestinal flora. *Evidence Based Complementary Alternative Medicine*.

[33] Zhao J. N., Zhang Y., Lan X. (2019). Efficacy and safety of xinnaoning capsule in treating chronic stable angina (qi stagnation and blood stasis syndrome). *Medicine*.

[34] Qiao L. J., Qi Z., Tu L. P., Zhang Y. H., Zhu L. P., Xu J. T. (2018). The association of radial artery pulse wave variables with the pulse wave velocity and echocardiographic parameters in hypertension. *Evidence Based Complementary Alternative Medicine*.

[35] Gomes Ribeiro Moura N., Sá Ferreira A. (2016). Pulse waveform analysis of Chinese pulse images and its association with disability in hypertension. *Journal of Acupuncture and Meridian Studies*.

[36] Wolff B., Macioce V., Vasseur V. (2020). Ten‐year outcomes of anti‐vascular endothelial growth factor treatment for neovascular age‐related macular disease: a single‐centre French study. *Clinical & Experimental Ophthalmology*.

[37] Komatsu T., Fujihara K., Yamada M. H., Sato T., Kitazawa M., Yamamoto M. (2020). 449-P: impact of body mass index (BMI) and waist circumference (WC) on coronary artery disease (CAD) in Japanese with and without diabetes mellitus (DM). *Diabetes*.

[38] Shi Y. L., Jiang T., Hu X. J., Cui J., Cui L. T., Tu L. P. A study on fatigue classification by logistic regression method: based on data of tongue and pulse. 2021, research square. https://www.researchsquare.com/article/rs-551999/v1.

